# Characteristics of Precipitation, Streamflow, and Sediment Transport of the Hangman Creek in the Pacific Northwest, USA: Implication for Agricultural Conservation Practice Implementation

**DOI:** 10.3390/hydrology12010003

**Published:** 2024-12-31

**Authors:** Yongping Yuan, Sean Kanyuk

**Affiliations:** 1 U.S. Environmental Protection Agency, Office of Research and Development, Research Triangle Park, NC 27711, USA; 2 Oak Ridge Institute for Science and Education (ORISE) Student Service Contract at U.S. Environmental Protection Agency, Office of Research and Development, Research Triangle Park, NC 27711, USA

**Keywords:** precipitation, streamflow, sediment transport, streamflow/precipitation ratio, high flow season, flow duration curve, agricultural conservation practices

## Abstract

Anthropogenic climate change and changes to land use and land management practices can have significant impacts on streamflow and sediment transport. In this study, we investigated long-term precipitation, streamflow, and suspended sediment load patterns within the Hangman Creek watershed, draining from the Rocky Mountains in Idaho to Washington, to identify the magnitude of changes with the goal of better understanding the links between these processes and the potential effects of agricultural conservation practices (ACPs) implemented since the 1990s. Comparing the study periods of 1991 to 2020 with 1961 to 1990, 1991 to 2020 had lower streamflow/precipitation ratios in the highest flow months such as February and March. Most streamflow occurred during winter and spring, so did suspended sediment. In addition, 2018 had much lower suspended sediment load compared to earlier years (1999 and 2000) during high flow seasons (January to April) given that streamflow was higher in 2018 than in 1999 and 2000. These changes may be attributed to the adoption of agricultural conservation practices because land cover remained almost unchanged from 2001 to 2021 and ACP adoption increased. Finally, the flow frequency analysis showed a strong linkage between higher streamflow events and increased suspended sediment load, with between 81% and 96% of total annual suspended sediment loads transported during the highest 10% of flows.

## 1. Introduction

Hangman Creek (HC), a major tributary to the Spokane River in Washington, experiences water quality problems including high sediment and phosphorus concentrations. It transports approximately 23% of the total phosphorus load to Lake Spokane on an annual basis, despite contributing only 3% of the total streamflow [1]. The phosphorus delivery from HC is mainly associated with suspended sediments and turbidity [2]. Therefore, HC is on Washington State’s list of impaired water bodies (the 303[d] list) for exceeding the limits for turbidity along with fecal coliform, pH, temperature, and dissolved oxygen. The Washington State Department of Ecology (WSDE) has developed a Total Maximum Daily Load (TMDL) that regulates the amount of sediment and phosphorus in HC. In addition, the draft Spokane River Dissolved Oxygen TMDL recommends limits on phosphorus loads coming from HC [2].

Sediment load, along with soil-bound elements (mainly phosphorous related to the TMDL in the Hangman Creek watershed (HCW)) to waterbodies, is dependent on the sediment transport capacity of the stream as well as the sediment quantity in the stream. Thus, understanding how water moves through a watershed after precipitation provides the foundation for understanding and describing how landscapes and water interact. The Hangman Creek watershed is located within the semi-arid region of the interior Pacific Northwest (PNW) east of the Cascades and west of the Rocky Mountains. This region is well known for having natural characteristics conducive to high soil erosion risk such as hilly topography, high percentages of silt loam soil texture, and a winter season that consists of both numerous freeze/thaw events and rain-on-snow events [3]. Additionally, large swaths of land in this region are utilized for dryland agriculture, which also increases risk of soil erosion. Agricultural practices such as tilling cropland and planting, monoculture cropping, excessive irrigation, and residue burning can result in disturbance of soils and degradation of soil health, which in turn makes the soil more susceptible to erosion [4–7].

Flooding is pervasive in the United States, with 99% of counties reporting at least one flooding event between 1996 and 2019 based on data provided by the Federal Emergency Management Agency (FEMA) [8]. In Spokane County, Washington, within the HCW, 36 flooding events were recorded during that time [8]. Flooding in this area is mainly caused by excessive moisture from precipitation events or subsequent rapid snow melt [9]. Understanding the characteristics of precipitation and streamflow response is crucial for developing effective control measures to mitigate flood damage and soil erosion.

Changes in climate can have significant effects on streamflow regime within a watershed [10]. A study incorporating climate data from 141 stations across the PNW region concluded that annual mean temperatures increased by 0.6 to 0.8 °C in the region between 1900 and 2012 [11]. Increases in temperature may result in changes in the hydrologic cycle, such as increasing the rate of evapotranspiration, decreasing snowfall, and potentially reducing the volume of surface runoff [12]. Using the Variable Infiltration Capacity model, it was found that streamflow in Idaho’s Salmon River decreased in spring and summer and increased in winter as air temperature increased [12]. Another study of the Spokane River watershed by Fu et al. [13] demonstrated that the relationship between streamflow, precipitation, and temperature is complex and non-linear: increasing precipitation might result in increased streamflow, while increasing temperature might result in decreasing streamflow. Similar studies, however, have not been performed in the HCW. In addition, none of these studies have addressed the potential implications for the soil erosion and total suspended sediment (TSS) load changes that have been a primary concern for the Spokane River. Finally, agricultural conservation practices (ACPs) have been increasingly implemented [14] and understanding their impacts on hydrology, soil erosion, and sediment loss is very important for the Conservation Effects Assessment Project (CEAP) program, a joint effort led by the USDA Natural Resources Conservation Service https://www.nrcs.usda.gov/ceap/faqs (accessed on 22 December 2024).

The overall goal of this study was therefore to analyze patterns of precipitation, streamflow, and sediment transport and their relationships to gain better understanding of their characteristics and interactions. We further explored the influence of the implemented agricultural conservation practices (ACPs) on these factors to potentially improve their effectiveness. For example, if implemented ACPs change the responses of streamflow to precipitation and/or the response of sediment transport to precipitation and streamflow, how can we better use that information to inform future ACP implementations? This study would therefore help us make more informed decisions about choosing suitable management practices to better manage water resources and mitigate soil erosion to achieve overall water quality goals. More specifically, our detailed objectives were: (1) to analyze historical precipitation and streamflow; (2) to assess the association between streamflow and sediment transport; and (3) to explore potential impacts of ACPs on streamflow and their effectiveness in reducing sediment loss.

## 2. Methods and Procedures

### 2.1. Study Area

**Figure 1.**
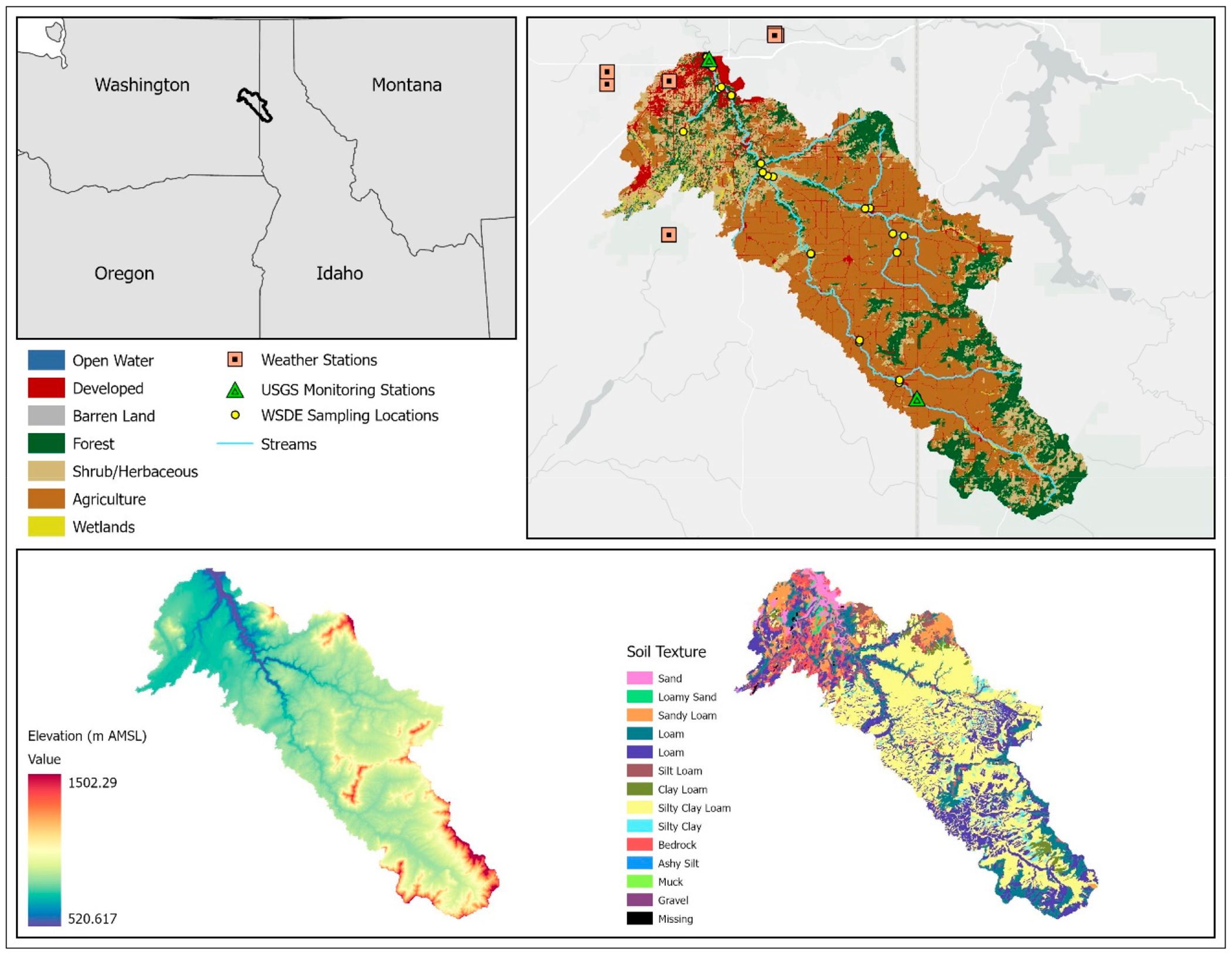
The Hangman Creek watershed and its location, major streams, meteorological observation stations, USGS/WSDE monitoring stations, elevation, and soil texture.

The Hangman Creek watershed (HCW) extends approximately 88 km from its headwaters in the foothills of the Rocky Mountains of Western Idaho to where the tributary empties into the Spokane River of Eastern Washington. The watershed encompasses an area of 174,508 ha. and contains roughly 354 km of perennial streams [15]. Some of the major tributaries that feed into HC include Rock Creek, California Creek, Little Hangman Creek, Marshall Creek, Spangle Creek, and Cove Creek. The headwaters of the basin lie at about 1100 m above sea level whereas at the outlet the elevation drops to as low as 520 m above sea level (Figure 1).

The dominant soil in the Hangman Creek watershed is silt clay loam, which accounts for about 44% of all soil types. Silt loam and loam soils represent 22% and 13% of soils, respectively (Table S1). These soils have moderate-to-low infiltration rates that contribute to the flashy flow regime observed in the HCW. The dominant hydrologic soil group type is Hydrologic Group soil C, covering 48% of the watershed area (Table S2, Figure 1).

The dominant land use in the watershed is agriculture (cultivated crops and pasture/hay), encompassing 52% (~93,564 ha) of the watershed area (Table 1), followed by forest and shrubland, which cover 19% (~33,367 ha) and 13% (~23,149 ha), respectively. Developed land only covers 8% (~13,792.05 ha) of the watershed, located within the northern part of the watershed around the city of Spokane (Figure 1).

### 2.2. Data Collection

This study included data sources covering land use/land cover, soils, precipitation, watershed boundary, stream discharge, and suspended sediment load used for HCW analysis (Table 2). In addition, land management practices such as planting and harvesting (Table S3 in Supplemental Materials) and ACP adoptions including conservation tillage and cover crops were also collected (Table 3) for Spokane County, where 68% of the watershed is located.

#### 2.2.1. Historic Precipitation, Temperature, and Snowfall

Daily and monthly precipitations were retrieved from the National Centers for Environmental Information (NCEI) Climate Data Online (CDO) and the National Weather Service (NWS) National Oceanic and Atmospheric Administration (NOAA) Online Weather Data (NOWData), respectively (Table 2). The weather station at the Spokane International Airport was the only source of available ground-based meteorological data located within the HCW. This station has a long history, spanning from August 1889 to the present, with 100% coverage of daily precipitation. There are stations outside the study area, such as Fairchild Airforce Base, Felts Field Airport, and Turnbull National Refuge, but these stations were not selected for use because they have patchy/short periods of record or are close to Spokane International Airport. As a result, data from Spokane International Airport were selected to act as proxy for daily precipitation conditions over the entire watershed. This station may not be fully representative of higher elevations found in the southern portion of the watershed that typically have higher precipitation and snowfall, but this slight regional difference could not be addressed due to a lack of available data in that area. Additionally, the monthly average temperature and monthly total snowfall data for the period 1961 to 2020 were retrieved from NOAA NOWData (Table 2).

#### 2.2.2. USGS Streamflow and Sediment

There are two United States Geological Survey (USGS) gauge stations located along HC (the main stem of the watershed) (Figure 1). At the upstream USGS Gauge 12422990, daily streamflow has been recorded since June 2007; whereas at the downstream USGS Gauge 12424000, daily streamflow has been recorded since April 1948. Daily suspended sediment load data were also collected from October 1998 to September 2001 at the downstream gauge. Thus, we decided to use the data from the downstream gauge, and the data from the upstream were not used due to its relatively short span of operation and lack of suspended sediment data.

#### 2.2.3. Monitoring Data from the Washington State Department of Ecology and Spokane Conservation District

The Washington State Department of Ecology (WSDE) has maintained permanent and temporary monitoring stations within the watershed. As part of a long-term statewide water quality monitoring program, the WSDE maintains a site near the outlet of the watershed (Hangman Creek at its mouth), where water quality samples have been collected and analyzed for such analytes as total suspended solids, nitrogen, and phosphorus on approximately a monthly basis since 1978 (Figure 1). In addition, as part of a field study investigating nutrient and sediment sources, the WSDE conducted a high-flow season study investigating sediment and phosphorus during the spring runoff season (Stuart, 2022). For that study, twenty-nine monitoring sites were set up and samples were collected on an approximately biweekly basis from January to May 2018 (Table S4 in Supplemental Materials).

### 2.3. Data Analysis of Precipitation, Streamflow, and Total Suspended Sediment

#### 2.3.1. Precipitation and Streamflow Characteristics, and Their Relationships

**Figure 2.**
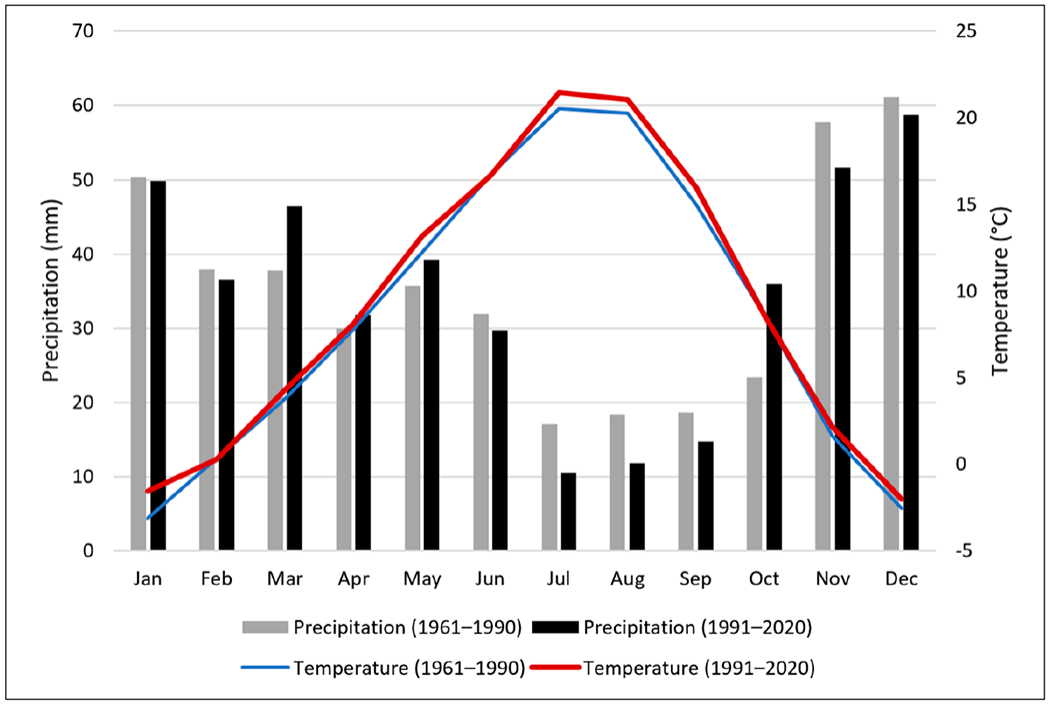
Comparisons of mean monthly precipitation and surface air temperature between the periods of 1961 to 1990 and 1991 to 2020.

To understand the characteristics of historical precipitation and potential changes, we compared two time periods, 1961 to 1990 vs. 1991 to 2020 (Figure 2). These two periods were based on water years as opposed to calendar years to allow for direct comparisons with annual streamflow, which is usually reported as water year by the USGS. For example, the 1961 water year spans from 1 October 1960 to 30 September 1961. Comparisons were made on 30-year averages (1961 to 1990 vs. 1991 to 2020) and monthly distribution (monthly averages of 1961 to 1990 and 1991 to 2020) (Figure 2).

**Figure 3.**
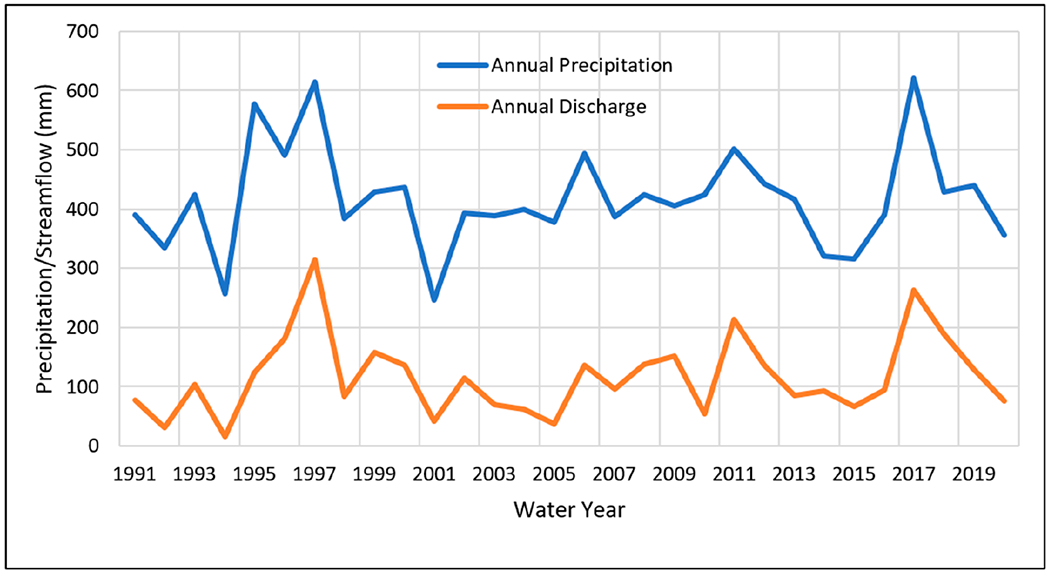
Annual precipitation vs. streamflow discharge in water years spanning from 1991 to 2020.

**Figure 4.**
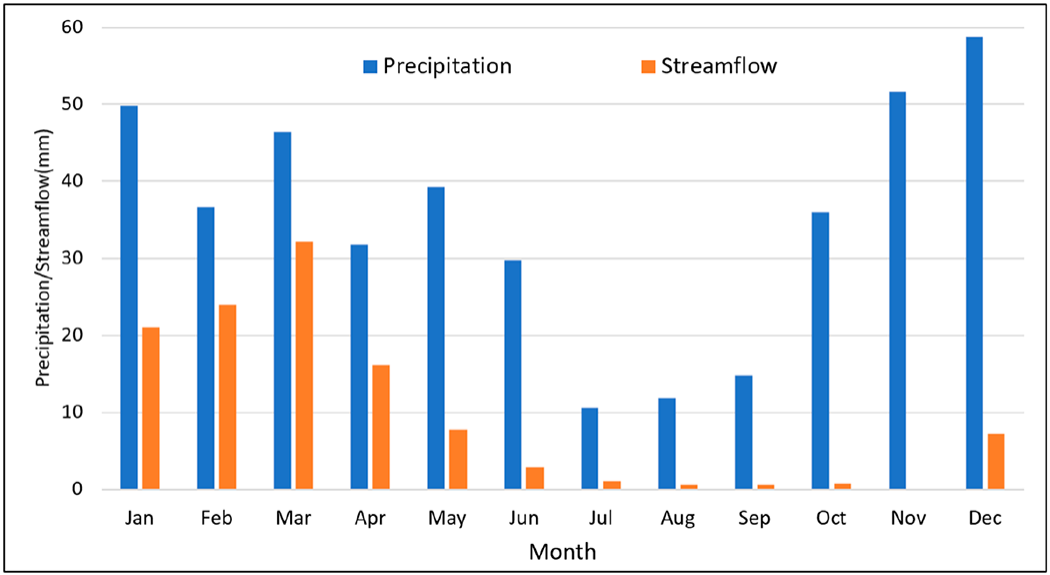
Average monthly precipitation and streamflow for the period of 1991 to 2020.

**Figure 5.**
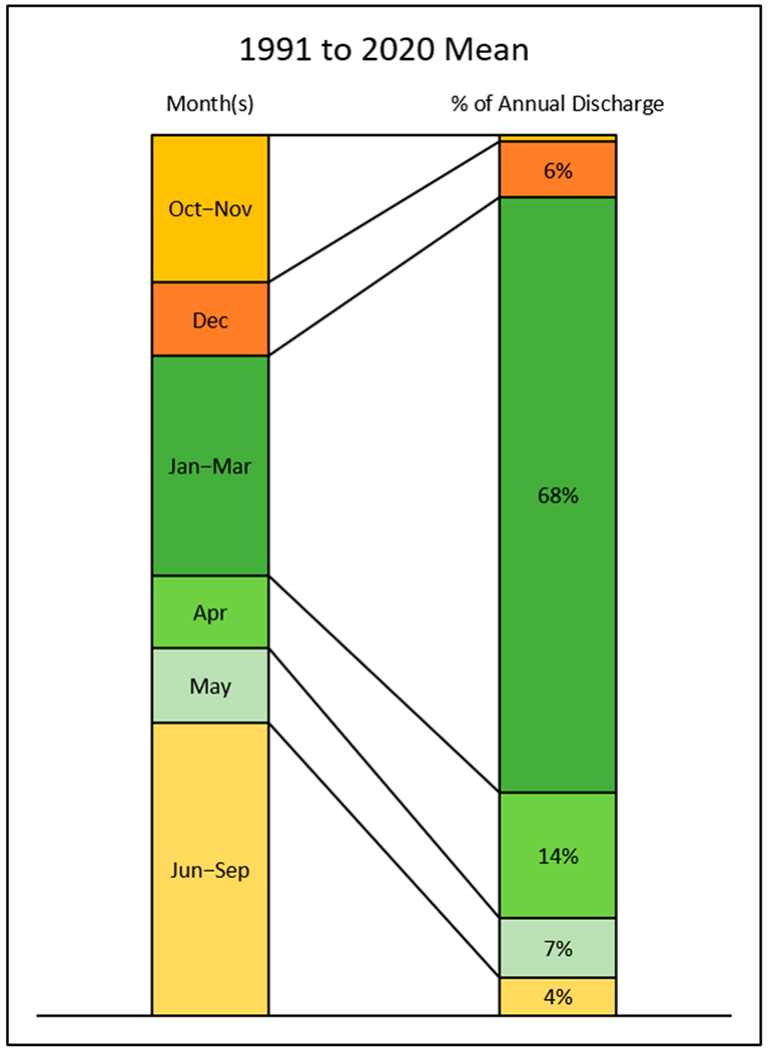
Monthly streamflow distribution on 30-year (1991 to 2020) average based on data observed at the USGS Gauge 12424000.

**Figure 6.**
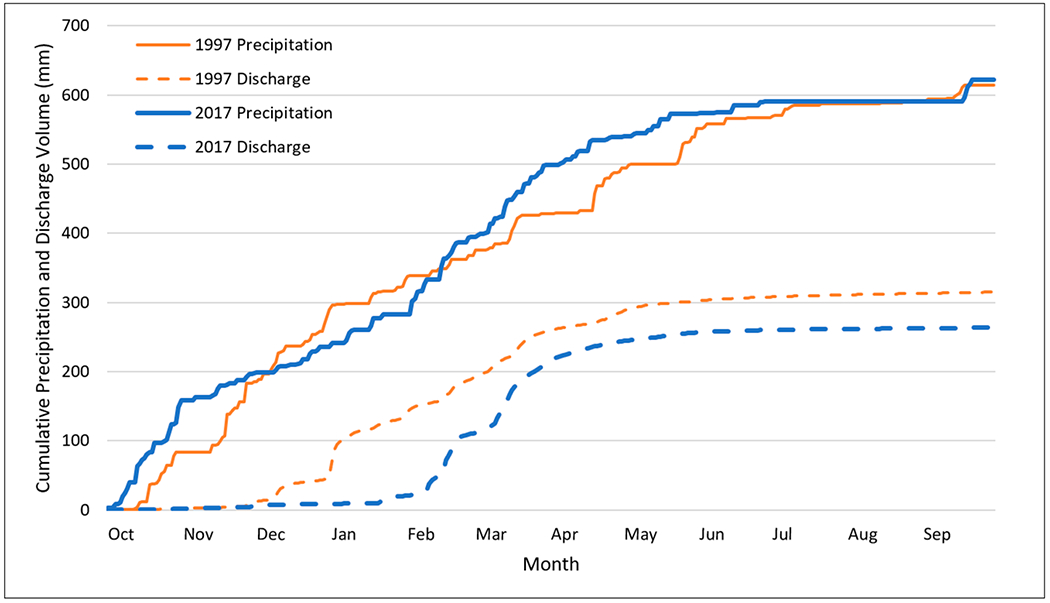
Accumulative precipitation and streamflow for 1997 and 2017.

To understand streamflow characteristics as well as response to precipitation, annual streamflow totals were obtained near the watershed outlet (USGS Gauge 12424000) and compared to annual precipitation (Figure 3). Monthly streamflow data were also obtained at the same gauge and compared to monthly precipitation for the period of 1991 to 2020 (Figure 4) as well as during two time periods (1961 to 1990 and 1991 to 2020). Further, monthly streamflow distribution was analyzed for each year, as well as the 30-year average from 1991 to 2020 to understand intra-annual variation (Figure 5). Finally, cumulative precipitation and streamflow was analyzed based on daily data for 1997 and 2017 (the two wettest years with precipitation much higher than the 30-year average) (Figure 6).

#### 2.3.2. Peak Flow Discharge

To provide better insight into the most extreme streamflow conditions within the HCW, annual peak streamflow measurements were collected from the downstream USGS Gauge 12424000 for the period 1991 to 2020. Once peak streamflow values and peak dates were identified, fifteen-minute streamflow observations were acquired from the gauge to determine reasonable beginning and end times for each annual peak flow event. Fifteen-minute data measurements were utilized to account for the flashy flow regime of HC. Daily streamflow data from the same station were utilized for events where fifteen-minute data were unavailable (1991, 1997, and 2009). The total flow volume during each peak flow event and duration of the event (elapsed time in hours) were also summarized. Since the occurrence of each peak flow event was a result of temperature and accumulation of rainfall and/or snowfall, it is very difficult to connect peak flows with precipitation events, and therefore total precipitation from the start of the water year was summarized.

#### 2.3.3. Flow Duration Curve and Sediment Load from 1999 to 2001

Daily flows in a given water year (1999 to 2001) were used to develop annual flow duration curves (USEPA, 2007) for each year (Figure S1 in Supplemental Materials). Briefly, daily flows were sorted in descending order, then categorized into five flow intervals: high flows (0–3rd percentile) as H, moist conditions (3–5th percentile) as M, mid-range flows (5–10th percentile) as MR, dry conditions (10–100th percentile) as U10, and low flows (90–100th percentile) as L (USEPA, 2007) as flow duration intervals were expressed as a percentage with zero corresponding to the highest daily discharge and 100 to the lowest (Table 4). Values provide breakpoints for high flows, moist conditions, and mid-range flows (Table S5, Supplemental Materials).

Flow interval loads were defined as the percentage of the annual loads released within each interval of the flow duration curve [16–18]. To calculate flow interval loads, daily suspended sediment loads were first summed for each breakpoint in the flow duration curve (i.e., 3th, 5th, 10th, and 100th percentiles) with the 100th percentile equivalent to the annual load, and then those breakpoint loads were converted to a percentage of the annual load. Flow interval loads were then calculated by subtracting the previous percentile from the next percentile (Table 4). For example, the moist condition load (M_3–5_) was calculated by subtracting the high flow load (M_3_%) from the upper 5% load (M_5_%). We analyzed flow interval loads to assess the significance of hydrology in driving sediment loss for each year.

In addition to developing flow duration curves for those three years (1999–2001), accumulative precipitation, streamflow, and sediment load were also analyzed using the daily data to gain further insights into these parameters and their relationships (responses). Sediment loads were converted from imperial tons/day to metric tonnes/day and then summed to obtain monthly and annual load.

### 2.4. Total Suspended Sedimen Load During High Flow Season

The 2018 WSDE high flow season study provided estimates of suspended sediment load at the mouth of Hangman Creek for two overlapping periods (18 January 2018 to 30 April 2018; and 1 March 2018 to 31 May 2018) [19]. Detained information on how total suspended sediment load was calculated can be found in the report [19]. To identify if any changes in total suspended sediment load occurred over years—more specifically, if any implemented land management practices/agricultural conservation practices (ACPs) reduced the total suspended sediment load—we summarized daily suspended load for the same periods (18 January to 30 April 2018, and 1 March to 31 May 2018) for the years of 1999, 2000, and 2001 when suspended sediment load data were available. To make the comparison more insightful, we also summarized daily precipitation and streamflow for those two periods for 1999, 2000, 2001, and 2018. In the 2018 WSDE high flow season study, average daily suspended sediment concentrations (SSCs) at the mouth of Hangman Creek were estimated for the same two study periods using two different regression models, which explains the differences for the overlapping periods [19].

## 3. Results and Discussion

### 3.1. Changes in Precipitation and Temperature

For the past 30 years from 1991 to 2020, annual precipitation ranged from 246 mm to 622 mm with an annual average of 417 mm, while for the period of 1961 to 1990, annual precipitation ranged from 251 mm to 585 mm with an annual average of 420 mm. Comparing the two 30-year climate study periods of 1961–1990 to 1991–2020 (Figure 2 and Table 5), there have been seasonal shifts in precipitation timing but the difference in total annual average precipitation is small (a slight decrease in the latter period). In general, precipitation decreased in summer (June to September) and winter (November to February of the next year) months, with larger decreases in July and August (about 7 mm or 37%) than those in winter months (Table 5), indicating a drier summer and potential summer drought. However, precipitation increased in spring (March to May), with a 9 mm (or 23%) increase in March, as well as in fall when October showed a 13 mm (or 54%) increase (Table 5).

For the past 30 years from 1991 to 2020, the hottest months were July/August and the temperature varied from 8 to 36 °C; the coldest months were December/January and the temperature varied from −15 to 9 °C. Comparing the two 30-year climate study periods of 1961–1990 to 1991–2020, the temperature was about the same in July/August while the temperature varied from −18 to 8 °C in December/January (slightly warmer in the latter period). Furthermore, the monthly average temperatures increased for nearly all months of the recent study period, with the highest increases occurring in January, July, and August (Figure 2 and Table S6 in Supplemental Materials), further indicating potential drought in the summer because a higher temperature would increase evapotranspiration in this time. The annual average temperature increased by 0.6 °C overall, and by about 1.0 °C in July and August, over the last 30-year period (Table S6 in Supplemental Materials).

Changes in climate patterns apparent in the HCW have been observed by other researchers in the region [11,20]. Snyder et al. [20] found that temperatures increased by 0.7 °C to 1.4 °C within the Columbia Plateau (which contains the Hangman Creek watershed) and Great Basin regions between 1985 and 2011. Abatzoglou et al. [11] also found that the annual mean temperature increased by 0.6 °C to 0.8 °C within the PNW region between 1900 and 2012 by analyzing data from 141 long-term climate data sites. Regarding precipitation changes, Abatzoglou et al. [11] also concluded that precipitation increased in the spring season and decreased in the summer season in a long-term trend as we observed in the HCW (Figure 2).

An analysis of snowfall during these two climate study periods shows that the annual average snowfall decreased by 54 mm or 5% in the period from 1991 to 2020 (Table 6). Snowfall decreased in six months of the year, with the highest decrease of 55 mm observed in January. The increased temperature in January (1.6 °C increase) may explain the snowfall decrease (Figure 2 and Table S6 in Supplemental Materials). Increases in snowfall only occurred in the months of February, March, and September, with the highest increase of 26 mm observed in February. For September, no snowfall was observed during the 1961-to-1990 period, but it was observed in September of 2019 during the 1991-to-2020 period. Increased air temperature (an 1.6 °C increase in January) would result in decreases in annual snowfall totals, which may result in higher runoff, and potentially increased sediment load, because decreases in annual snowfall mean more rainfall events given that the annual average precipitation remained almost unchanged (a 3 mm increase on annual average). Furthermore, higher temperatures could mean a faster snow melt and therefore a higher streamflow pulse to streams, which means increased flood potential.

### 3.2. Connecting Precipitation to Streamflow Characteristics

#### 3.2.1. Annual Precipitation and Streamflow from 1991 to 2020 and 1961 to 1990

The changes in annual streamflow generally reflected the changes in annual precipitation as expected (Figure 3), but with a few exceptions such as 2010 (424 mm precipitation), when there was relatively lower streamflow compared to other years. Precipitation varied from 246 mm to 622 mm during the period 1991 to 2020 (Table 7). The response of the streamflow to precipitation varied year to year, with a streamflow-to-precipitation ratio ranging from 0.06 in the second-driest year (1994) with a precipitation of 257 mm to 0.51 in the second-wettest year (1997), with a precipitation of 615mm (Table 7). The average annual precipitation during the 30-year period from 1991 to 2020 was 417 mm, and the 30-year average annual streamflow was 116 mm, with a streamflow/precipitation ratio of 0.28 on annual average (Table 7). In 2010, the precipitation was 424 mm, slightly above the annual average (417 mm), but streamflow was only 54 mm, with a streamflow/precipitation ratio of 0.13. The driest year was 2001, with a precipitation of 246 mm, while the wettest year was 2017, with a precipitation of 622 mm (Table 7). The wettest year (622 mm in 2017) produced the second-highest streamflow (263 mm), with a streamflow/precipitation ratio of 0.42; whereas the driest year (246 mm in 2001) produced a streamflow of 42 mm and a streamflow/precipitation ratio of 0.17 (Table 7 and Figure 3).

Similar patterns were observed for the years of 1961 to 1990, although the annual average streamflow was slightly lower (5 mm) during this period (Table S7 in Supplemental Materials). The responses of streamflow to precipitation were also similar: the 30-year average annual streamflow was 111 mm, with a streamflow/precipitation ratio of 0.26 (Table S7 in Supplemental Materials).

One of the objectives of this study was to see if the implemented ACPs have changed the responses of streamflow to precipitation; more specifically, if the implemented ACPs have increased infiltration, and thus decreased streamflow. We cannot corroborate this from annual streamflow changes because the annual streamflow/precipitation ratio increased from 0.26 (Table S7 in Supplemental Materials) to 0.28 (Table 7 and Figure 3) when comparing the two study periods (1961 to 1990 vs. 1991 to 2020). Reduced snowfall (54 mm decrease annually) and earlier spring snow melt may have contributed to this increased streamflow/precipitation ratio. Just as demonstrated by other researchers [12,13], the relationship between streamflow, precipitation, and temperature is complex and non-linear; increased rainfall due to decreased snowfall as observed in the HCW (Table 6) might result in increased streamflow, which might have concealed any benefit of ACP implementation on an annual basis.

#### 3.2.2. Monthly Precipitation and Streamflow

The average monthly streamflow/precipitation ratio varied throughout the year during the years 1991–2020 (Figure 4). Higher ratios were observed in the winter and spring months compared to the summer and fall months (Figure 4). Most of the streamflow occurred from December to May. The lowest streamflow occurred in November, with a streamflow/precipitation ratio of less than 1% (Figure 4). A similar pattern of streamflow/precipitation ratios was observed for the 1961 to 1990 period, when most streamflow occurred from December to May (Figure S2 in Supplemental Materials). Comparing the monthly streamflow/precipitation ratios between the two periods (1961 to 1990 vs. 1991 to 2020), 1991 to 2020 had lower ratios during the highest flow months, such as February and March (Figure S2 in Supplemental Materials), indicating decreased streamflow during the highest flow months for the same amount of precipitation (February and March). This is important when talking about flood mitigation and reducing suspended sediment transport. One of the factors contributing to lower streamflow in the highest flow months in the latter period could be the adoption of ACPs after the 1990s, including the conversion of conventional tillage to conservation tillage, direct seeding to reduce soil disturbance, the installation of grassed waterways, and the construction of water and sediment control structures at 14 sites within the HCW between 2010 and 2014 [14].

#### 3.2.3. Streamflow Distribution and High Flow/Low Flow Periods 1991 to 2020

The majority of streamflow occurred during the winter and spring (December to May) months (Figure 4). On a 30-year average, 95% of streamflow occurred during December to May, while only 5% occurred during June to November (Figure 5). Thus, studies conducted by the WSDE classified flow in the high flow period, which encompasses the winter and spring months, and in the low flow period, which encompasses the summer and fall months [19]. A further analysis of monthly streamflow distribution used the intervals of October through November and June through September to represent the lowest streamflow periods and the interval of January through March to represent the highest streamflow (Table S8 in Supplemental Materials). The months of December, April, and May were separated into individual intervals due to the highly variable nature of annual streamflow observed in these months (Table S8 in Supplemental Materials).

Starting in October, more precipitation occurred and increased from October through December, when it reached a plateau (Figure 4). However, little streamflow occurred in October and November (the lowest streamflow/precipitation ratio in November, Figure 4). From December to March, the streamflow/precipitation ratio increased until it plateaued in March (Figure 4).

On the 30-year (1991 to 2020) average, the flow from Jan to March is about 68% of the total annual flow and 27% occurred during the rest of winter and spring months (Figure 5). The flow from Jan to March ranged from 41% (2005) to 86% (1992) (Table S8 in Supplemental Materials). In the two wettest years of 1997 and 2017, the flow from January to March was about 69% and 80% of the annual flow (Figures S3 and S4 in Supplemental Materials), respectively.

The unique seasonality of streamflow and sediment transport in PNW watersheds brings challenges for water resource management, such as drought and flood mitigation as well as water quality improvement (e.g., reducing soil erosion and sediment load to the Spokane River). On the 30-year (1991 to 2020) average (Figure 4), precipitation was the lowest in the summer months, such as July to September, when crops need water the most, indicating the necessity of irrigation for crop production in this area. In addition to irrigating as needed, increasing soil moisture when water is available potentially decreases the need for irrigation. Therefore, any ACPs which promote infiltration and soil moisture might be beneficial to crop production and should be encouraged. Another challenge is finding management practices that can be applied to increase infiltration in winter since about 68% of the annual flow occurred during January to March.

#### 3.2.4. Daily Streamflow and Precipitation Comparison for the Two Wettest Water Years

The total annual precipitation in 1997 (615 mm) was about the same as that in 2017 (622 mm), but the total streamflow in 2017 (263 mm) was much lower than that in 1997 (315 mm) (Table 7). Although the reasons for a lower streamflow/precipitation ratio in 2017 could be complicated, land use and land management changes such as the implementation of ACPs might be one of the reasons which contributed to a lower streamflow in 2017. Plots of accumulative precipitation/streamflow for those two years show that streamflow started to increase at the end of December in 1997, while streamflow did not start until late February in 2017 (Figure 6). Throughout the year, accumulated streamflow remained lower in 2017 than in 1997, regardless of the changes in accumulated precipitations between 1997 and 2017 (i.e., a higher accumulated precipitation was observed in 2017 until middle December, when accumulated precipitation in 1997 exceeded that of 2017; and accumulated precipitation in 2017 exceeded 1997 around February and remained higher until July). Streamflow was much reduced by the end of April for both years. The streamflow is almost zero from May to December (Figure 6).

In general, less precipitation occurred from December to February in 2017 compared to the same period in 1997 (Figure 6). In addition, a higher snowfall was observed in 1997 (2045 mm) compared to that in 2017 (1562 mm), which may correspond to a higher precipitation from December to February in 1997 (Figure 6). Less snowfall may result in a higher streamflow based on Tang et al. (2012). Thus, management practice changes may also contribute to the lower streamflow in 2017, since land use/land cover from the National Land Cover Database (NLCD) in 2001 and 2021 remained relatively static over the past two decades.

#### 3.2.5. Peak Flow Discharge

**Figure 7.**
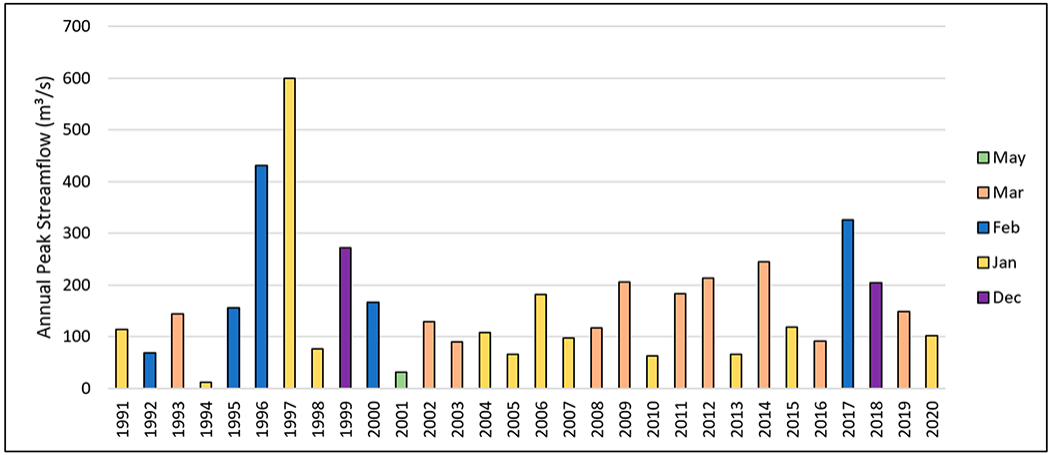
Peak streamflow values and the corresponding months they occurred at the USGS monitoring site (USGS ID 12424000) by water year.

Annual peak discharges mainly occurred during the wintertime (January to March) with a few events in December and May, which corresponded to the high flow season (Table 8). The water year 1997 had the highest peak discharge, which occurred on 1 January, followed by 1996, when the peak occurred on 8 February, and then 2017, when the peak was on 17 February (Figure 7). The later and lower peak discharge in 2017 can be attributed to a lower precipitation in December and January compared to 1997. In fact, there was not much precipitation in December and January 2017, as previously discussed (Figure 6). The total accumulated precipitation was 296 mm when the peak happened in 1997, whereas the total accumulated precipitation was 364 mm when the peak happened in 2007 (Table 8). Although the total accumulated precipitation was higher (364 mm) in 2017 than 1997 (296 mm) before the peak occurred, the peak in 1997 was nearly double the value in 2017 (600 m^3^/s vs. 326 m^3^/s). Furthermore, the total flow volume produced from the peak event was higher in 1997 than 2017 (71 mm vs. 63 mm).

Peak flow events lasted between 96 and 647 h and produced streamflow as high as 71 mm in 1997, which was about 23% of annual total flow that year (24% of annual total flow in 2007). Streamflow produced from peak flow events contributed between 6% and 39% of the annual total flow during the 30-year period of 1991 and 2020 (Table 8).

Reducing the peak and total flow volumes produced during peak events is key to reducing flooding risk. Any practices increasing the roughness of landscape and channels, as well as increasing infiltration, would help to reduce flood risk and sediment transport. Finding practices promoting infiltration and reducing peak values/total flow amount under frozen ground, however, would be very challenging. An intensive literature search did not yield any results for addressing this issue. Future research should focus on this topic due to the climate conditions in the PNW.

### 3.3. Precipitation, Streamflow, and Sediment from 1999 to 2001

**Figure 8.**
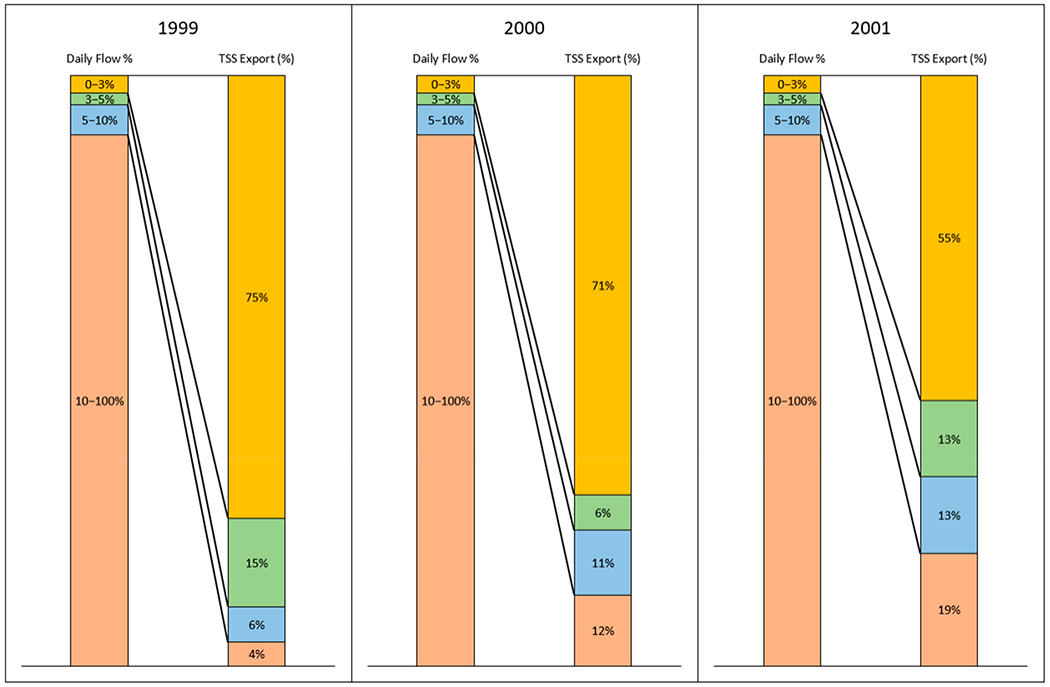
Flow frequency and the percentage of annual sediment load mobilized during flow intervals for the 1999, 2000, and 2001 water years.

Since daily suspended sediment load data were collected from October 1998 to September 2001 at the USGS gauge station 12424000, the daily flow and sediment data for those three years were used to develop annual flow duration curves [21] (Supplemental Materials). In general, suspended sediment loads were predominantly transported during high flow periods (Figure 8). The top 3% of flows transported more than 50% of the annual suspended sediment load, especially in 1999, when 75% of the annual suspended sediment load was transported (Figure 8). The top 10% flow transported 96%, 88%, and 81% sediment load for 1999, 2000, and 2001, respectively (Figure 8). The remaining 90% of flows transported only 4%, 12%, and 19% of the annual suspended sediment loads for 1999, 2000, and 2001, respectively (Figure 8).

**Figure 9.**
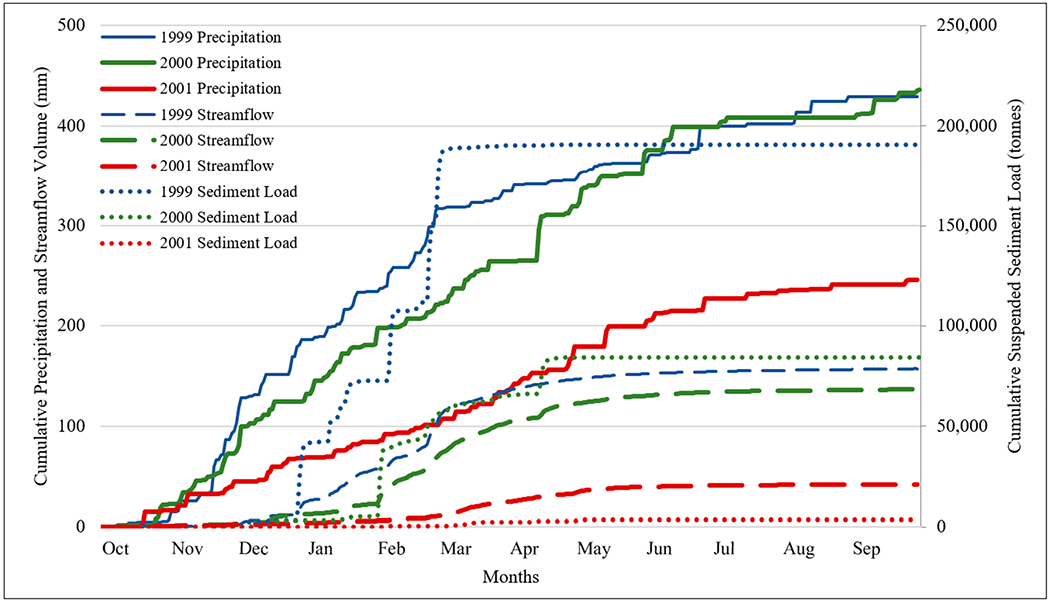
Annual accumulation of precipitation, streamflow, and sediment loss for 1999, 2000, and 2001 water years.

The precipitation for these three years was 430 mm, 436 mm, and 246 mm, respectively (Table 7), with the 2001 precipitation below the 30-year average (417 mm). As a matter of fact, 2001 was the driest year, with only 246 mm precipitation for all years from 1991 to 2020. Comparing 1999 and 2000, 1999 had a slightly higher annual flow (157 mm) than 2000 (137 mm), although 2000 had a slightly higher annual precipitation (436 mm vs. 430 mm) (Table 7). The annual suspended sediment load, however, was much greater in 1999 compared to that in 2000 (190,731 tonnes vs. 84,405 tonnes) (Figure 9). The year 1999 had a much higher peak discharge (271 m^3^/s) than 2000 (167 m^3^/s) (Table 8), which may have resulted in a much higher sediment loss in 1999. Since 2001 was the driest year with only 246 mm precipitation, the TSS was correspondingly low (Figure 9).

Unlike many other agricultural regions where soil erosion and sediment loss are more associated with agricultural activities, such as tilling and planting [22,23], the strong seasonality of precipitation and runoff in the PNW leads to most of the sediment loss occurring during the dormant seasons and perhaps on frozen ground (Figure 9) when there are no agricultural activities (Table S3 in Supplemental Materials). In 1999, the year with the highest sediment load, 100% of the sediment was delivered from December to March (Figure S5 in Supplemental Materials). In 2000, 100% of the TSS was delivered from December to April (Figure S5 in Supplemental Materials). In 2001, major sediment losses occurred from December to April, when 74% of the TSS was delivered (Figure 9 and Figure S5 in Supplemental Materials). This seasonal pattern of sediment loss associated with peak flows presents challenges for reducing soil erosion and sediment loss, since the majority of ACPs for water quality improvements focus on reducing soil disturbance and maintaining ground cover.

### 3.4. Total Suspended Sediment Load During High Flow Season

#### 3.4.1. Comparison of High Flow Season Sediment Loads from 2018 vs. 1999 to 2001

One of the challenges during this study was a lack of data, particularly a lack of consistent sediment data. The 2018 WSDE high flow season study collected sediment samples during January to May and subsequently estimated the suspended sediment load at the mouth of Hangman Creek for two overlapping periods (18 January 2018 to 30 April 2018 and 1 March 2018 to 31 May 2018) based on two regression models [19] (Table 9). Details on why two regression models were used can be found in the report [19].

The total suspended sediment load was estimated to be 28,032 tonnes in the January-to-April period in 2018 from the high flow season study conducted by the WSDE (Table 9) [19]. However, the total suspended sediment load was 116,265 tonnes in 1999 and 71,527 tonnes in 2000 for the same period from those three years of monitoring by the USGS. Comparing precipitation and streamflow for that same period, 2018 showed 156 mm and 111 mm, respectively, while 1999 showed 139 mm and 106 mm, respectively, and 2000 showed 147 mm and 101 mm, respectively. Although the precipitation and streamflow were a little higher in 2018 than 1999 and 2000 for the same period, the suspended sediment load was much lower in 2018 than 1999 and 2000. The total suspended load was the lowest in 2001 because that year had much less precipitation (86 mm) and streamflow (36 mm) compared to others.

In the March-to-May time period, the total suspended sediment load was estimated to be 19,196 tonnes in 2018 [19], 13,418 tonnes in 1999, and 24,657 tonnes in 2000 for those three years of monitoring by the USGS. For that same monthly time frame, 2018 showed a precipitation of 121 mm and streamflow of 77 mm, while 1999 showed a precipitation of 47 mm and streamflow of 45 mm, and 2000 showed a precipitation of 152 mm and streamflow of 58 (Table 9). Although the streamflow was higher in 2018 than in 2000, the suspended sediment load was much lower in 2018 than in 2000. The total suspended load was lower in 1999 than in 2018 due to lower precipitation and lower streamflow in 1999 than 2018.

#### 3.4.2. Implications for ACP Implementation for Sediment Control

Higher precipitation and streamflow, but lower sediment loads, in the 2018 water year suggest that changes in land management practices within the watershed that occurred over the years might be the reason for the lower observed sediment loads. For the total period from 18 January to 31 May, the estimated sediment load ranged from 32,594 to 34,500 tonnes for 2018 due to estimation uncertainty [19], while the sediment loads were 116,350 tonnes and 71,579 tonnes for 1999 and 2000, respectively (Table 9)—approximately 2–3 times higher than the load in 2018. A review of previous work completed in the watershed shows that best management and/or ACPs have been implemented over the years, and examples of reported actions include the conversion of conventional tillage to conservation tillage (about 2316 ha), direct seeding to reduce soil disturbance (about 1215 ha), the installation of grassed waterways, and the construction of water and sediment control structures at 14 sites [14].

Edge-of-field studies, conducted between 2019 and 2021, demonstrated the effectiveness of conservation tillage and no-tillage on reducing sediment loss [24]. This three-year edge-of-field monitoring study showed that sediment loss in the no-tillage field was significantly lower than from the conventional tilled field (0.82 lbs. ac^1^ yr^−1^ vs. 167 lbs. ac^1^ yr^−1^) [24]. Based on the increased adoption of agricultural conservation practices and the proven effectiveness of some of these practices in reducing soil erosion and sediment loss in the region, it is reasonable to attribute the decreased sediment loads observed in 2018 to the implementation of ACPs.

Analysis of land use/land cover from NLCD 2001 and 2021 showed that land use/land cover of the watershed remained relatively static over the past two decades. Nearly all land cover types changed by less than 1%, apart from the evergreen forest land cover, which decreased by 1.36%. Cultivated cropland slightly decreased (0.03%), while hay/pasture slightly increased (0.08%) and resulted in a slight increase in agricultural land use (the total of cultivated cropland and hay/pasture) (Table S9 in Supplemental Materials). Therefore, the impact of land use/land cover changes on watershed hydrology, soil erosion, and sediment loss should be minor or negligible overall, which suggested that the decrease in suspended sediment loads observed in 2018 could likely be attributed to the results of the implementation of ACPs. Conversion of conventional tillage to direct seeding could potentially reduce soil erosion and sediment loss.

## 4. Conclusions and Recommendations

This study analyzed precipitation, streamflow, and suspended sediment data available in the HCW to explore the characteristics of precipitation, streamflow, and sediment transport and their relationships as well as possible changes over the years. Comparing the two 30-year climate periods of 1961–1990 and 1991–2020 indicated decreased precipitation and increased temperatures with drier summers. Annual streamflow/precipitation ratio increased from 0.26 to 0.28 between the two 30-year periods, indicating a higher streamflow was produced in the latter study period on an annual average, potentially resulting from a decreased snowfall and earlier spring snow melt. However, the monthly streamflow/precipitation ratio was lower for the highest flow months, such as February and March, in the more recent study period of 1991 to 2020 compared to 1961 to 1990, indicating a decreased streamflow during the highest flow months (February and March). An analysis of monthly streamflow distribution revealed that 95% of streamflow occurred from December to May and only 5% occurred from June to November on a 30-year average. Further analysis of the two wettest years (1997 and 2017) revealed that 2017 had a lower streamflow/precipitation ratio compared to 1997 (0.42 vs. 0.51). A lower monthly streamflow/precipitation ratio in the highest flow months during 1991 to 2020 compared to 1961 to 1990, as well as a lower streamflow/precipitation ratio in 2017 compared to 1997, suggests that changes in land management practices within the watershed might have contributed to this lower streamflow/precipitation ratio. Similar to monthly streamflow distribution, the majority of sediment was delivered during winter months, from December to April. In addition, the top 10% of streamflow transported 88% of annual sediment based on data from 1999 to 2001. Comparing TSS from the high flow season in 2018 to 1999 and 2000, 2018 had a much lower TSS load than that in 1999 and 2000, although the precipitation and streamflow were higher in 2018 from January to April. This suggests that changes in land management practices over the study years might be the reason for lower TSS in 2018. Future studies should focus on documenting detailed ACP adoption, including the type of practices, when, where, and how much. In addition, how those practices impact water quality should also be better documented through more water quality monitoring efforts.

## Supplementary Material

Supplement1

## Figures and Tables

**Table 1.** Land use/land cover of the Hangman Creek watershed derived from the 2021 National Land Cover Database (NLCD).

NLCD Land Cover Type	NLCD Class Number	Percent Area Covered	Total Area (ha.)
Open Water	11	0.1	158.8
Developed, Open Space	21	2.9	5259.1
Developed, Low Intensity	22	3.0	5430.2
Developed, Medium Intensity	23	1.4	2534.6
Developed, High Intensity	24	0.3	568.3
Barren Land	31	0.1	116.7
Deciduous Forest	41	0.0	47.5
Evergreen Forest	42	18.5	33,218.6
Mixed Forest	43	0.1	100.4
Shrub/Scrub	52	12.9	23,148.8
Herbaceous	71	6.8	12,238.6
Hay/Pasture	81	2.3	4059.7
Cultivated Crops	82	49.9	89,504.5
Woody Wetlands	90	0.8	1339.7
Emergent Herbaceous Wetlands	95	0.9	1678.6

**Table 2.** Summary of existing datasets used in this study.

Data Elements	Origin	Source
Soil characteristics	USDA Natural Resources Conservation Service (NRCS)	gSSURGO version 2.4 (available at: https://www.nrcs.usda.gov/resources/data-and-reports/gridded-soil-survey-geographic-gssurgo-database) (accessed on 22 December 2024).
Land cover	USGS Earth Resources Observation and Science (EROS) Center	NLCD 2021 Land Cover (CONUS) (available at: https://www.mrlc.gov/data?f[0]=year:2021) (accessed on 22 December 2024).
Precipitation (Daily)	NOAA National Centers for Environmental Information (NCEI)	Daily Summaries (available at: https://www.ncei.noaa.gov/cdo-web/search?datasetid=GHCND) (accessed on 22 December 2024).
Precipitation (Monthly)	NWS NOAA Online Weather Data (NOWData)	Monthly summarized data (available at: https://www.weather.gov/wrh/climate?wfo=otx) (accessed on 22 December 2024).
Temperature (Monthly)	NWS NOAA Online Weather Data (NOWData)	Monthly summarized data (available at: https://www.weather.gov/wrh/climate?wfo=otx) (accessed on 22 December 2024).
Snowfall (Daily)	NOAA National Centers for Environmental Information (NCEI)	Daily Summaries (available at: https://www.ncei.noaa.gov/cdo-web/search?datasetid=GHCND) (accessed on 22 December 2024).
Snowfall (Monthly)	NWS NOAA Online Weather Data (NOWData)	Monthly summarized data (available at: https://www.weather.gov/wrh/climate?wfo=otx)
Hangman Creek Watershed boundary	USGS National Hydrography Dataset (NHD)	Watershed Boundary Dataset (WBD) (available at: https://www.usgs.gov/national-hydrography/access-national-hydrography-products) (accessed on 22 December 2024).
Stream discharge (Daily)	USGS National Water Information System (NWIS)	Daily data (available at: https://waterdata.usgs.gov/nwis/inventory?site_no=12424000&agency_cd=USGS) (accessed on 22 December 2024).
Stream discharge (Monthly)	USGS National Water Information System (NWIS)	Monthly statistics (available at: https://waterdata.usgs.gov/nwis/inventory?site_no=12424000&agency_cd=USGS) (accessed on 22 December 2024).
Peak discharge	USGS National Water Information System (NWIS)	Peak discharge (available at: https://waterdata.usgs.gov/nwis/inventory?site_no=12424000&agency_cd=USGS) (accessed on 22 December 2024).
Suspended sediment load	USGS National Water Information System (NWIS)	Daily data (available at: https://waterdata.usgs.gov/nwis/inventory?site_no=12424000&agency_cd=USGS) (accessed on 22 December 2024).
Suspended sediment load	Washington State Department of Ecology	2018 High flow study (available at: https://apps.ecology.wa.gov/publications/SummaryPages/2203004.html) (accessed on 22 December 2024).
Agricultural conservation practices	USDA	(2012, 2017, and 2022) Census of Agriculture: Washington State and County Data Reports (available at: https://www.nass.usda.gov/AgCensus/) (accessed on 22 December 2024).
Agricultural management practices	USDA Natural Resources Conservation Service	WEPS NRCS Crop Management Zone 47 Template (available at: https://www.nrcs.usda.gov/crop-management-templates) (accessed on 22 December 2024).

**Table 3.** Implemented agricultural conservation practices for Spokane County, where 68% of the watershed is located.

Year	No-Till		Reduced Tillage		Conventional Tillage		Cover Cropping	
	# of Farms	Total Area (ha)	# of Farms	Total Area (ha)	# of Farms	Total Area (ha)	# of Farms	Total Area (ha)
2012	168	37,193	164	39,449	371	25,351	83	781
2017	209	46,218	135	40,569	217	18,176	92	1303
2022	281	43,821	203	49,410	384	18,834	168	1863
10-Year Change	+113	+6628	+39	+9970	+13	−6517	+85	+1082

**Table 4.** Description of flow intervals and associated sediment transport.

Flow Interval	Abbreviation	Flow Interval	Suspended Sediment Load Exported
High Flows	H	Q ≥ Q_3_	M_3%_
Moist Conditions	M	Q_3_ ≥ Q ≥ Q_5_	M_3–5_
Upper 5% Flows	U5	Q ≥ Q_5_	M_5%_
Mid-Range Flows	MR	Q5 ≥ Q ≥ Q_10_	M_5–10_
Upper 10% Flows	U10	Q ≥ Q_10_	M_10%_
Low Flows	L	Q_10_ ≥ Q ≥ Q_100_	M_10–100_

**Table 5.** Mean monthly precipitation (mm) observed during the two climate periods of 1961–1990 and 1991–2020 based on observations from the Spokane International Airport. Red = increase, Green = decrease.

Study Period	Jan	Feb	Mar	Apr	May	Jun	Jul	Aug	Sep	Oct	Nov	Dec	Annual
1961–1990	50.34	37.91	37.81	30.01	35.74	31.90	17.10	18.41	18.65	23.39	57.74	61.19	420.21
1991–2020	49.87	36.60	46.42	31.76	39.23	29.70	10.52	11.85	14.80	35.99	51.67	58.68	417.11
Change	−0.47	−1.31	8.61	1.74	3.50	−2.20	−6.58	−6.55	−3.85	12.60	−6.07	−2.51	−3.10
% Change	−0.94	−3.46	22.77	5.81	9.78	−6.90	−38.47	−35.60	−20.65	53.85	−10.51	−4.10	−0.74

**Table 6.** Mean monthly snowfall depth (mm snow–water equivalent) observed during the two climate periods of 1961–1990 and 1991–2020 based on observations from the Spokane International Airport. Red = increase, Green = decrease.

Study Period	Jan	Feb	Mar	Apr	May	Jun	Jul	Aug	Sep	Oct	Nov	Dec	Annual
1961–1990	360.34	171.37	92.03	22.44	4.32	0.00	0.00	0.00	0.00	8.28	163.15	379.31	1201.50
1991–2020	305.39	197.44	103.63	17.44	2.12	0.00	0.00	0.00	2.79	5.00	156.17	362.29	1147.06
Change	−54.95	26.08	11.60	−5.00	−2.20	0.00	0.00	0.00	2.79	−3.56	−6.99	−17.02	−54.44

**Table 7.** Annual precipitation and streamflow for the water years from 1991 to 2020.

Water Year	Annual Precipitation (mm) *	Annual Snowfall (mm)	Annual Streamflow (mm)	Streamflow/Precipitation Ratio (%)
1991	391	1072	77	20
1992	335	470	31	9
1993	425	2217	104	24
1994	257	500	16	6
1995	578	757	124	21
1996	491	1019	182	37
1997	615	2045	315	51
1998	384	465	83	22
1999	430	1080	157	37
2000	436	1046	137	31
2001	246	1234	42	17
2002	393	1626	115	29
2003	388	538	69	18
2004	399	1389	62	16
2005	378	655	37	10
2006	494	739	136	28
2007	387	904	96	25
2008	424	2352	137	32
2009	405	2482	152	38
2010	424	366	54	13
2011	502	1753	213	42
2012	443	935	137	31
2013	416	1105	86	21
2014	320	955	94	29
2015	316	447	67	21
2016	390	869	94	24
2017	622	1562	263	42
2018	429	1252	189	44
2019	440	1445	129	29
2020	356	1133	76	21
Total	12,513	34,412	3475	789
30-Year Average	417	1147	116	28

* Precipitation summarized in this table is for water years; e.g., the precipitation for 1991 is from October 1st 1990 to September 30 of 1991 to be compatible with the water year defined by the USGS for streamflow.

**Table 8.** Streamflow observed during annual peak streamflow events for the water years spanning from 1991 to 2020 compared to the total annual streamflow volume.

Water Year	Date of Peak	Peak Discharge (m^3^/s)	Total Streamflow (mm)	Elapsed Time (h)	% Annual Streamflow	Precipitation (mm)
1991	13 January 1991	113.27	22.88 *	408.00 *	30.07	182.12
1992	21 February 1992	68.24	8.82	132.00	29.39	211.58
1993	15 March 1993	143.85	11.08	107.75	10.71	215.14
1994	5 January 1994	11.19	1.70	204.00	10.96	101.09
1995	20 February 1995	156.03	18.86	215.75	15.36	277.88
1996	8 February 1996	430.42	51.91	263.25	28.82	227.33
1997	1 January 1997	600.32	70.70 *	360.00 *	23.10	295.66
1998	28 January 1998	76.46	7.86	156.00	9.80	170.94
1999	28 December 1998	271.28	14.23	179.50	9.13	180.85
2000	3 February 2000	166.79	20.33	204.00	15.06	197.61
2001	1 May 2001	31.43	5.14	418.75	12.62	179.58
2002	12 March 2002	129.41	15.92	267.75	14.02	238.76
2003	23 March 2003	89.76	8.71	156.00	12.70	272.03
2004	30 January 2004	107.89	10.11	191.75	16.35	143.00
2005	19 January 2005	65.70	3.42	95.75	9.53	116.33
2006	11 January 2006	181.79	38.25	348.25	28.30	215.39
2007	4 January 2007	97.41	8.05	156.00	8.54	199.64
2008	12 March 2008	116.95	47.07	647.00	34.45	275.08
2009	8 January 2009	206.43	18.72 *	384.00 *	12.38	180.34
2010	6 January 2010	63.43	4.84	143.75	9.17	159.26
2011	10 March 2011	182.93	17.98	142.75	8.49	322.07
2012	31 March 2012	212.94	29.24	188.75	21.56	292.61
2013	26 January 2013	65.13	4.96	132.00	5.97	223.77
2014	6 June 2014	254.22	19.72	155.00	21.34	171.96
2015	18 January 2015	118.08	8.82	192.25	13.22	164.34
2016	23 March 2016	91.18	22.44	456.00	23.92	324.36
2017	17 February 2017	325.64	62.70	335.75	24.10	363.47
2018	30 December 2017	204.16	14.33	119.75	7.81	181.86
2019	24 March 2019	148.10	46.32	468.00	36.50	276.61
2020	25 January 2020	101.94	29.28	396.00	38.84	172.47

* Values were calculated using daily streamflow due to missing data in 15 min streamflow.

**Table 9.** Streamflow, precipitation, and sediment loads recorded for the periods of January 18th to April 30th and March 1st to May 31st for the water years of 1999, 2000, 2001, and 2018.

January 18–April 30						
Water Year	Parameters	Jan	Feb	Mar	Apr	Entire Period
1999	Streamflow (mm)	15.98	50.00	28.78	10.97	105.73
	Precipitation (mm)	18.03	83.06	17.53	11.18	129.79
	Sediment Load (tonnes)	11,024.05	91,908.22	12,916.43	416.40	116,265.10

2000	Streamflow (mm)	0.94	49.86	31.44	18.67	100.92
	Precipitation (mm)	9.91	40.89	41.66	54.86	147.32
	Sediment Load (tonnes)	812.55	46,109.96	7710.76	16,893.32	71,526.60

2001	Streamflow (mm)	4.91	4.72	13.97	10.97	34.57
	Precipitation (mm)	8.64	16.76	17.53	43.43	86.36
	Sediment Load (tonnes)	6.52	199.53	1754.85	313.16	2274.06

2018	Streamflow (mm)	21.87	22.39	36.76	30.00	111.01
	Precipitation (mm)	30.99	40.64	33.02	51.56	156.21
	Sediment Load (tonnes)	3810	7620	8437	8165	28,031.86
March 1–May 31						
Water Year	Parameters	Mar	Apr	May	Entire Period	

1999	Streamflow (mm)	28.78	10.97	5.37	45.12	
	Precipitation (mm)	17.53	11.18	18.54	47.24	
	Sediment Load (tonnes)	12,916.43	416.40	85.00	13,417.83	

2000	Streamflow (mm)	31.44	18.67	7.75	57.87	
	Precipitation (mm)	41.66	54.86	56.39	152.91	
	Sediment Load (tonnes)	7710.76	16,893.32	52.61	24,656.69	

2001	Streamflow (mm)	13.97	10.97	6.37	31.31	
	Precipitation (mm)	17.53	43.43	20.07	81.03	
	Sediment Load (tonnes)	1754.85	313.16	671.38	2739.38	

2018	Streamflow (mm)	36.76	30.00	10.22	76.97	
	Precipitation (mm)	33.02	51.56	36.83	121.41	
	Sediment Load (tonnes)	6468.19	6259.54	6468.19	19,195.93	

## Data Availability

See Table 2 on the data used for this study.
