# Supplementary material for: Characteristics of Precipitation, Streamflow, and Sediment Transport of the Hangman Creek in the Pacific Northwest, USA: Implication for Agricultural Conservation Practice Implementation
Source: Hydrology. Author manuscript; Available in PMC 2025 Dec 31. (PMC11980776; doi:10.3390/hydrology12010003)
Supplement: Supplement1 [file NIHMS2054896-supplement-Supplement1.zip › hydrology-3367813-supplementary.pdf]

Characteristics of precipitation, streamflow and sediment transport of the Hangman Creek in the Pacific Northwest USA: implication for agricultural conservation practice implementation

Yongping Yuan[a*] and Sean Kanyuk[b]

[a]US Environmental Protection Agency, Office of Research and Development, Research Triangle Park, NC 27711

[b]Oak Ridge (ORAU) Student Service Contract at US Environmental Protection Agency, Office of Research and Development, Research Triangle Park, NC 27711

[*]Corresponding author: Yongping Yuan, yuan.yongping@epa.gov, 109 T.W. Alexander Dr. RTP, NC 27711

Supplemental Materials

List of abbreviations

Acronym	Definition
ACP/BMP	Agricultural Conservation Practice/Best Management Practice
CDO	Climate Data Online
FEMA	Federal Emergency Management Agency
HC	Hangman Creek
HCW	Hangman Creek watershed
NLCD	National Land Cover Database
NOAA	National Oceanic and Atmospheric Administration
NCEI	National Centers for Environmental Information
NWS	National Weather Service
PNW	Pacific Northwest
TMDL	Total Maximum Daily Load
TSS	Total Suspended Sediment
USA	United States of America
USEPA	United States Environmental Protection Agency
USGS	United States Geological Survey
WSDE	Washington State Department of Ecology

Methods and procedures

Table S1: Surface Soil Texture (top 2-meter) in Hangman Creek watershed.

Soil Texture	Percent Area Covered	Total Area (ha.)
Sand	2.54	4,554.63
Loamy Sand	0.71	1,270.35
Sandy Loam	5.54	9,945.99
Loam	13.32	23,894.82
Silt Loam	22.40	40,191.21
Sandy Clay Loam	1.99	3,575.88
Clay Loam	1.74	3,129.84
Silty Clay Loam	44.13	79,164.72
Bedrock	5.07	9,088.2
Silty Clay	2.10	3,760.92
Muck	0.15	274.86
Gravel	0.03	46.17
Ashy Silt	0.02	28.53
Missing	0.27	479.97

Table S2: Soil Hydrologic Groups (based on top 2-meter) in Hangman Creek watershed.

Soil Hydrologic Group	Percent Area Covered	Total Area (ha.)
Group A	3.63	6,511.95
Group B	23.36	41,908.86
Group C	48.48	86,971.77
Group D	3.37	6,053.76
Group B/D	5.25	9,412.29
Group C/D	15.62	28,021.41
Missing	0.29	526.05

Table S3: Management practices associated with soil disturbance applied on major crop plantation types.

Rotation	Date	Management
**Winter Wheat (CT), Early Plant**		
Year 1	09/16	Tillage (Moldboard Plow)
	09/17	Tillage (Tandem Disk)
	09/19	Fertilizer Application
	09/20	Winter Wheat Planting (Drill or Air Seeder)
Year 2	05/15	Herbicide Application
	06/15	Fungicide Application
	07/01	Fungicide Application
	08/15	Harvest & Kill (Winter Wheat)
**Winter Canola-Winter Wheat (NT)**		
Year 1	10/01	Herbicide Application

Year 2	10/02	Spring Canola Planting
	05/05	Herbicide Application
	06/01	Insecticide Application
	08/15	Harvest and Kill (Winter Canola)
	09/15	Herbicide Application
	09/16	Fertilizer Application
	09/17	Winter Wheat Planting
Year 3	05/15	Herbicide Application
	06/15	Fungicide Application
	07/01	Fungicide Application
	08/15	Harvest & Kill (Winter Wheat)
**Winter Canola-Winter Wheat-Fallow (CT)**		
Year 1	05/01	Tillage (Spike Point Field Cultivator, Coil Tine Harrow)
	06/30	Tillage (Spike Point Field Cultivator, Coil Tine Harrow)
	08/01	Fertilizer Application
	08/05	Tillage (Spike Point Field Cultivator, Coil Tine Harrow)
	08/30	Winter Canola Planting
Year 2	05/05	Herbicide Application
	06/15	Insecticide Application
	08/15	Harvest & Kill (Winter Canola)
	09/15	Tillage (Offset Heavy Disk)
	09/16	Tillage (Spike Point Field Cultivator, Coil Tine Harrow)
	09/17	Tillage (Spike Point Field Cultivator, Coil Tine Harrow)
	09/18	Fertilizer Application
	09/19	Tillage (Spike Point Field Cultivator, Coil Tine Harrow)
	09/20	Planting Winter Wheat
Year 3	05/15	Herbicide Application
	06/15	Fungicide Application
	07/01	Fungicide Application
	08/15	Harvest & Kill (Winter Wheat)
	10/30	Tillage (Chisel, Twisted Shovel, Coil Tine Harrow)
**Lentil-Winter Wheat-Winter Wheat (CT)**		
Year 1	04/20	Tillage (Spike Point Field Cultivator, Coil Tine Harrow)
	04/21	Fertilizer Application
	04/25	Lentil Planting
	04/26	Soil Compacting (Cultipacker)
	05/19	Herbicide Application
	08/15	Harvest & Kill (Lentils)
	09/15	Tillage (Offset Heavy Disk)
	09/16	Tillage (Spike Point Field Cultivator, Coil Tine Harrow)
	09/17	Tillage (Spike Point Field Cultivator, Coil Tine Harrow)
	09/18	Fertilizer Application
	09/19	Tillage (Spike Point Field Cultivator, Coil Tine Harrow)
	09/20	Winter Wheat Planting
Year 2	05/15	Herbicide Application
	06/15	Fungicide Application

	07/01	Fungicide Application
	08/15	Harvest & Kill (Winter Wheat)
	10/11	Tillage (Chisel)
	10/12	Tillage (Spike Point Field Cultivator, Coil Tine Harrow)
	10/13	Fertilizer Application
	10/14	Tillage (Spike Point Field Cultivator, Coil Tine Harrow)
	10/15	Winter Wheat Planting
	05/15	Herbicide Application
	06/15	Fungicide Application
Year 3	07/01	Fungicide Application
	08/15	Harvest & Kill (Winter Wheat)
	10/01	Tillage (Moldboard Plow)

Flow duration curves

Flow duration curves were calculated for each water year with available data.

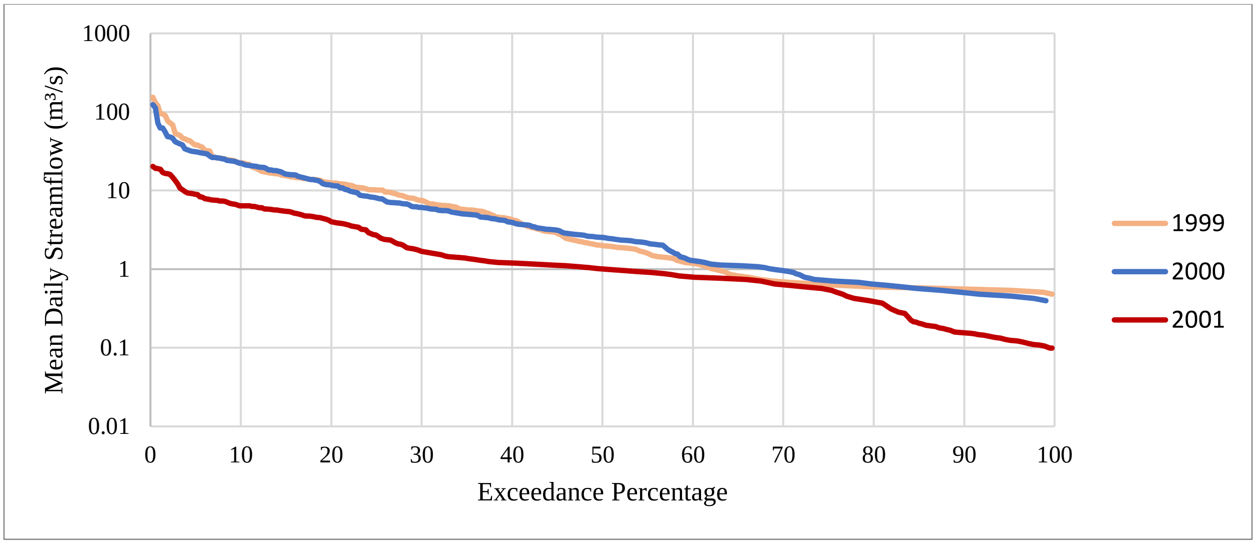

Figure S1: Flow Duration Curves for each year.

Table S4: Sample locations during high-flow study conducted by the Washington State Department of Ecology.

Location ID	Location Name	Latitude	Longitude
56HAN-58.5	Hangman Ck. at State Line	47.2028	-117.0406
56HAN-55.1	Hangman Ck. above Little Hangman Ck.	47.222	-117.0755
56LIT-00.1	Little Hangman Ck. at Connell St	47.2254	-117.0747
56HAN-47.0	Hangman Ck. at Marsh Rd.	47.2761	-117.1532
56COV-00.2	Cove Ck. at mouth	47.2788	-117.1531
56HAN-32.8	Hangman Ck. at Bradshaw Rd.	47.3928	-117.2481
56RAT-00.1	Rattler Run Ck. at mouth	47.3935	-117.2483
56ROC-19.6	Rock Ck. at Bradshaw Rd.	47.395	-117.0798
56ROS-00.4	Rose Ck. at mouth	47.4169	-117.0667
56ROC-17.1	Rock Ck. at Chatcholet Rd.	47.4201	-117.0883
56MIC-00.2	Mica Ck. at mouth	47.454	-117.1328
56ROC-13.0	Rock Ck. at Hwy 27 in Rockford	47.4532	-117.1422
56ROC-00.5	Rock Ck. at mouth	47.4955	-117.3228
56HAN-20.2	Hangman Ck. blw Rock Ck.	47.4961	-117.3337
56SPA-00.0	Spangle Ck. at mouth	47.5011	-117.3435
56CAL-00.1	California Ck. at mouth	47.5127	-117.3469
56HAN-06.2	Hangman Ck. at Meadowlane Rd.	47.603	-117.4058
56MIN-00.5	Minnie Ck. at mouth	47.5544	-117.4999
56MAR-00.4	Marshall Ck. at Qualchan Dr.	47.612	-117.4308
56MAR-00.0	Marshall Ck. at mouth	47.6141	-117.4253
56GAR-00.2	Garden Springs at Fish Lake trail	47.6443	-117.4509
56HAN-00.7	Hangman Ck. at mouth	47.6549	-117.4554
56MS4-Chestnut	Stormwater outfall at Chestnut St., US RB	47.6402	-117.443
56MS4-11thAve	Stormwater outfall at 11th Ave., DS RB	47.6458	-117.4473
56MS4-I90RB1	Stormwater outfall 100' US of I-90, RB	47.6485	-117.4461
56MS4-I90RB2	Stormwater outfall directly underneath I-90, RB	47.6488	-117.4463
56MS4-I90LB	Stormwater outfall 40' US of I-90, LB	47.6485	-117.4465
56CSO-19	CSO #19 outfall DS of I-90, RB	47.6493	-117.4464
56MS4-Sunset	Stormwater outfall DS Sunset Blvd. High Bridge Pk.	47.6503	-117.4487
56MS4-A-St	Stormwater outfall at A St. & Riverside Ave, US LB	47.6541	-117.454

Table S5: Suspended sediment loads reaching the outlet of the watershed during each of the five flow intervals during the water years of 1999 to 2001.

**1999**		
**Flow Interval**	**Sediment Load (tonnes)**	**Percentage of Annual Sediment Load**
**H**	142,643.26	74.79
**M**	28,019.61	14.69
**U5**	170,662.87	89.49
**MR**	12,430.36	6.52
**U10**	183,093.23	96.00
**L**	7,619.95	4.00
**2000**		
**Flow Interval**	**Sediment Load (tonnes)**	**Percentage of Annual Sediment Load**
**H**	59,906.31	70.98
**M**	5,007.09	5.93
**U5**	64,913.40	76.91
**MR**	9,489.91	11.24
**U10**	74,403.31	88.15
**L**	10,001.28	11.85
**2001**		
**Flow Interval**	**Sediment Load (tonnes)**	**Percentage of Annual Sediment Load**
**H**	1,899.00	54.51
**M**	452.14	12.98
**U5**	2,351.14	67.49
**MR**	466.37	13.39
**U10**	2,817.51	80.87
**L**	666.27	19.13

Results and discussion

Table S6. Mean monthly temperature observed during the two climate periods of 1961-1990 and 1991-2020 based on observations from the Spokane International Airport.

Study Period	Jan	Feb	Mar	Apr	May	Jun	Jul	Aug	Sep	Oct	Nov	Dec	Annual
**1961-1990**	-3.10	0.33	3.76	7.80	12.25	16.73	20.51	20.29	15.01	8.56	1.62	-2.57	8.43
**1991-2020**	-1.54	0.28	4.27	8.12	13.17	16.64	21.47	21.06	15.98	8.56	2.17	-2.04	9.01
**Change**	1.56	-0.05	0.51	0.31	0.92	-0.08	0.95	0.78	0.97	0.00	0.56	0.53	0.58
**% Change**	50.21	-15.82	13.53	4.01	7.53	-0.50	4.65	3.82	6.48	0.04	34.32	20.69	6.88

Table S7. Annual precipitation and streamflow for the period of 1961 to 1990.

**Water Year**	**Annual Precipitation (mm)** [a]	**Annual Snowfall (mm)**	**Annual Streamflow (mm)**	**Streamflow/Precipitation Ratio (%)**
**1961**	495	851	139	28
**1962**	450	1,704	73	16
**1963**	390	457	92	23
**1964**	483	1,608	88	18
**1965**	497	2,075	195	39
**1966**	284	1,128	66	23
**1967**	411	767	113	27
**1968**	324	754	56	17
**1969**	456	1,969	189	42
**1970**	386	1,013	126	33
**1971**	479	810	117	24
**1972**	400	1,704	157	39
**1973**	260	406	40	16
**1974**	524	1,427	274	52
**1975**	506	2,261	147	29
**1976**	417	1,240	132	32
**1977**	251	414	14	5
**1978**	586	1,763	100	17
**1979**	336	1,542	116	34
**1980**	389	973	58	15
**1981**	405	361	90	22
**1982**	409	1,204	148	36
**1983**	515	930	142	28
**1984**	488	1,214	181	37
**1985**	360	1,697	98	27
**1986**	410	1,552	110	27
**1987**	383	653	48	13
**1988**	423	856	29	7
**1989**	425	1,679	109	26
**1990**	463	1,034	90	20
**Total**	12,606	36,045	3,336	773
**30-Year Average**	420	1,202	111	26

[a] Precipitation summarized in this table are for water years, e.g. the precipitation for 1961 is from October 1st 1960 to September 30 of 1961 to be compatible with the water year defined by the USGS for streamflow.

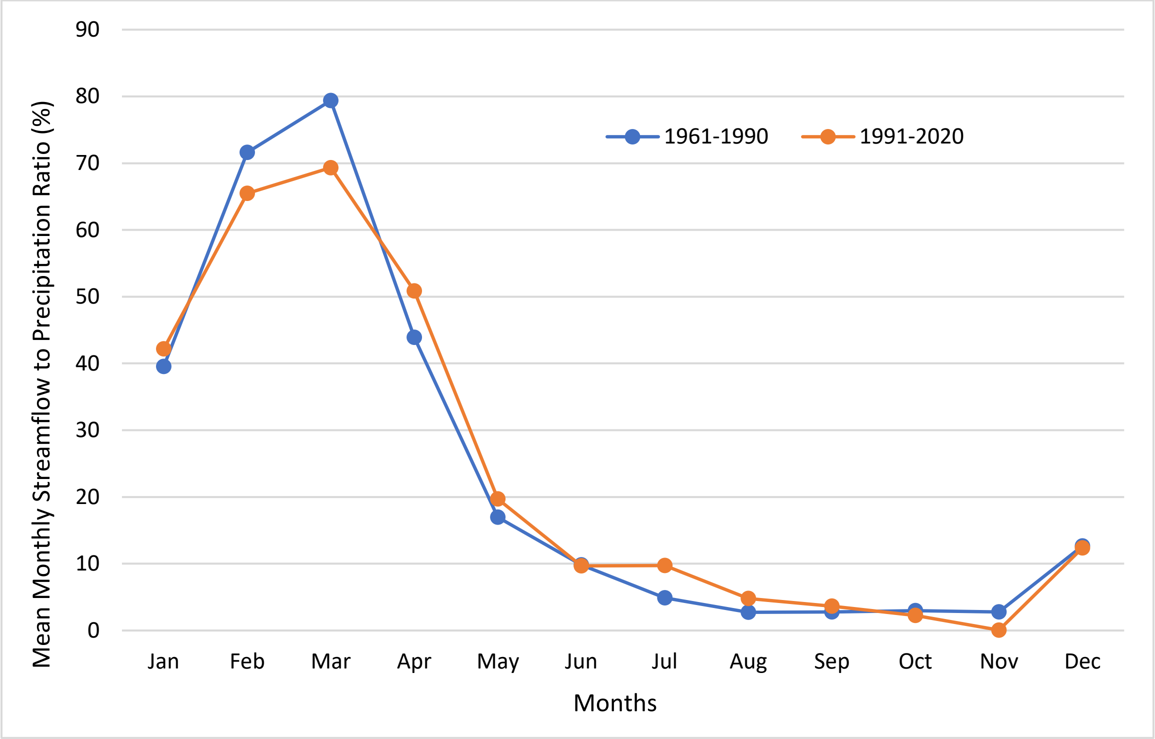

Figure S2. Mean monthly streamflow precipitation ratio (%) for the periods of 1961 to 1990 and 1991 to 2020.

Table S8: Discharge distribution for water years from 1991 to 2020 represented as the percentage of the annual streamflow.

Year	Oct-Nov	Dec	Jan-Mar	April	May	June-Sep
1991	1	4	75	9	7	4
1992	1	5	86	5	2	2
1993	0	0	64	19	13	4
1994	3	10	59	15	8	6
1995	0	14	74	7	3	3
1996	1	6	68	14	7	4
1997	1	12	69	9	5	4
1998	3	5	64	9	12	8
1999	1	14	71	7	3	3
2000	1	7	68	14	6	5
2001	3	4	51	21	16	6
2002	0	8	70	14	4	3
2003	1	2	76	12	7	3
2004	1	2	70	6	14	6
2005	2	7	41	19	25	7
2006	0	6	71	15	3	5
2007	1	10	75	9	3	3
2008	0	3	61	22	9	5
2009	0	1	64	25	6	3
2010	2	7	51	10	11	19
2011	0	6	58	18	13	5
2012	1	1	56	25	10	7
2013	1	10	67	14	4	4
2014	1	3	78	11	5	3
2015	1	7	75	12	3	2
2016	0	2	82	10	3	2
2017	1	1	80	11	5	3
2018	1	12	62	16	6	4
2019	1	5	55	29	5	4
2020	2	3	70	8	10	7
Average	1	6	68	14	7	4

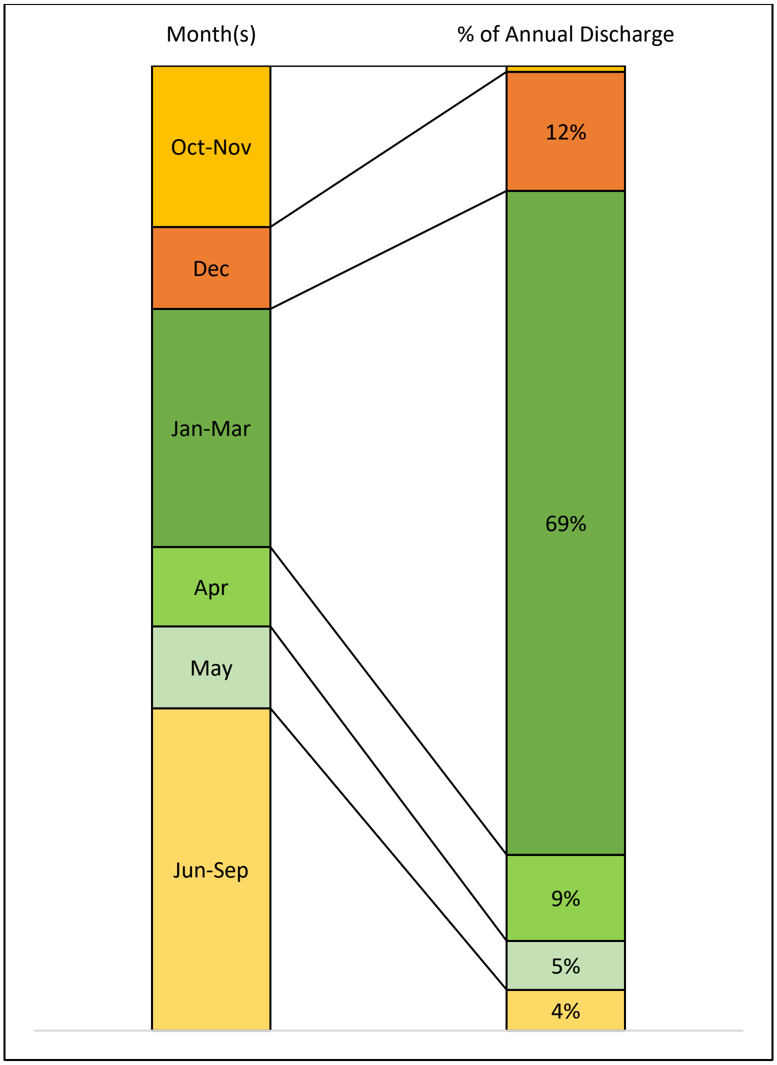

Figure S3: Monthly streamflow distribution for the 1997 water year observed at USGS Gauge 12424000.

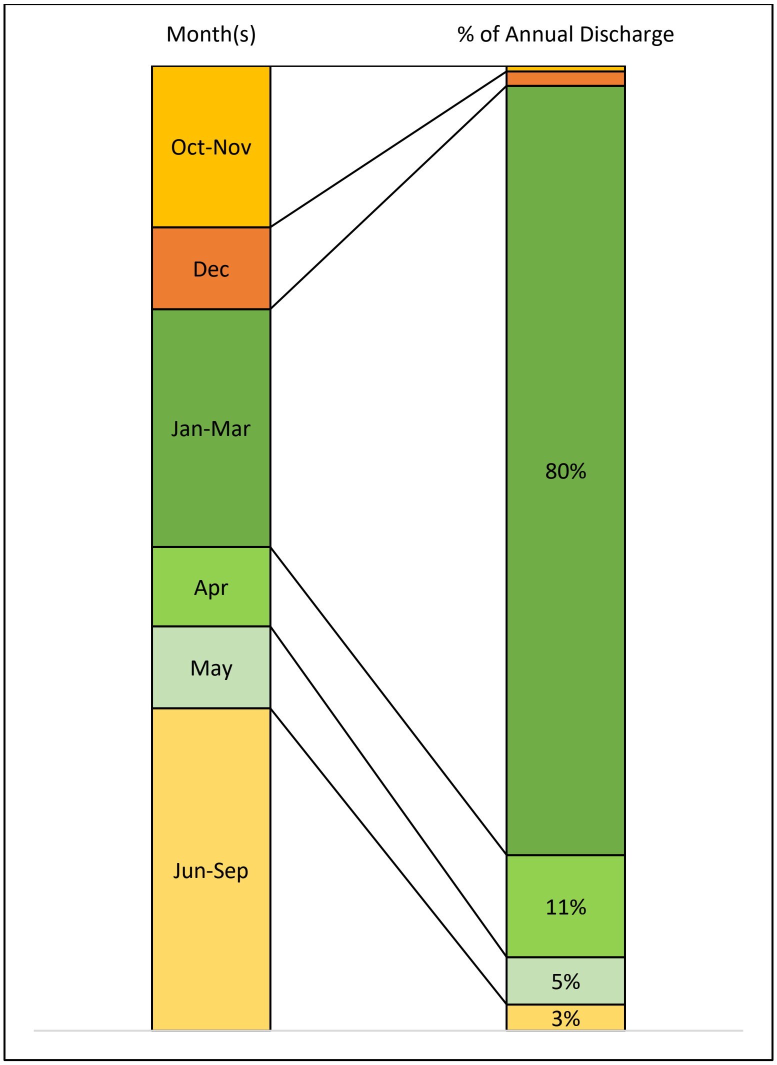

Figure S4: Monthly streamflow distribution for the 2007 water year observed at USGS Gauge 12424000.

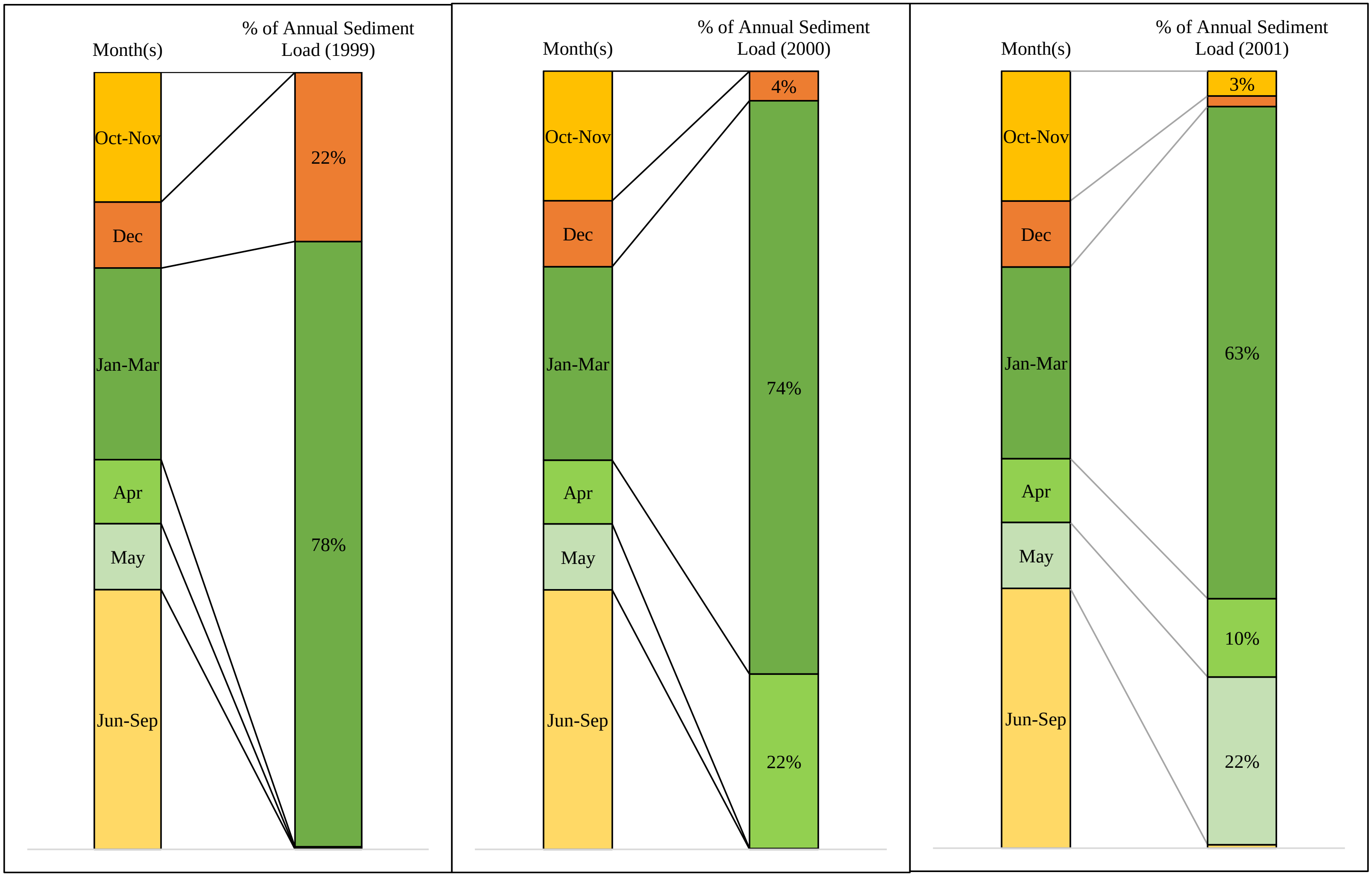

Figure S5. Monthly suspended sediment load distribution for 1999, 2000, and 2001 water year observed at USGS Gauge 12424000.

Table S9. Land use change comparison of the Hangman Creek Watershed based on the 2001 and 2021 NLCDs.

	2001		2021		Change	
NLCD Land Cover Type	Percentage	Area (ha.)	Percentage	Area (ha.)	Percentage	Area (ha.)
Open Water	0.089446201	160.47	0.088493045	158.76	0.00	-1.71
Developed, Open Space	3.004098071	5389.47	2.931407355	5259.06	-0.07	-130.41
Developed, Low Intensity	2.843365806	5101.11	3.026773159	5430.15	0.18	329.04
Developed, Medium Intensity	0.947838768	1700.46	1.412778416	2534.58	0.46	834.12
Developed, High Intensity	0.19665121	352.8	0.316748914	568.26	0.12	215.46
Barren Land	0.096369126	172.89	0.065065464	116.73	-0.03	-56.16
Deciduous Forest	0.002859469	5.13	0.026487714	47.52	0.02	42.39
Evergreen Forest	19.87797592	35661.87	18.51611612	33218.64	-1.36	-2,443.23
Mixed Forest	0.004464785	8.01	0.055985396	100.44	0.05	92.43
Shrub/Scrub	12.63313463	22664.34	12.90317888	23148.81	0.27	484.47
Herbaceous	6.528469025	11712.33	6.821790359	12238.56	0.29	526.23
Hay/Pasture	2.180119466	3911.22	2.262893572	4059.72	0.08	148.5
Cultivated Crops	49.9149935	89549.46	49.88991044	89504.46	-0.03	-45.00
Woody Wetlands	0.728612801	1307.16	0.746722772	1339.65	0.02	32.49
Emergent Herbaceous Wetlands	0.951601227	1707.21	0.9356484	1678.59	-0.02	-28.62
